# Video-Assisted Thoracoscopy for Vertebral Body Tethering of Juvenile and Adolescent Idiopathic Scoliosis: Tips and Tricks of Surgical Multidisciplinary Management

**DOI:** 10.3390/children9010074

**Published:** 2022-01-05

**Authors:** Sara Costanzo, Andrea Pansini, Luca Colombo, Valentina Caretti, Petar Popovic, Giulia Lanfranchi, Anna Camporesi, Gloria Pelizzo

**Affiliations:** 1 Pediatric Surgery Department, “Vittore Buzzi” Children’s Hospital, 20154 Milano, Italy; andrea.pansini@asst-fbf-sacco.it (A.P.); petar.popovic@asst-fbf-sacco.it (P.P.); giulia.lanfranchi@asst-fbf-sacco.it (G.L.); gloriapelizzo@gmail.com (G.P.); 2 Pediatric Orthopedics and Traumatology, “Vittore Buzzi” Children’s Hospital, 20154 Milano, Italy; luca.colombo@asst-fbf-sacco.it (L.C.); valentina.caretti@asst-fbf-sacco.it (V.C.); 3 Pediatric Anesthesia and Intensive Care Unit, “Vittore Buzzi” Children’s Hospital, 20154 Milano, Italy; anna.camporesi@asst-fbf-sacco.it; 4 Department of Biomedical and Clinical Science “L. Sacco”, University of Milano, 20157 Milano, Italy

**Keywords:** juvenile and adolescent idiopathic scoliosis, video-assisted thoracoscopy, anterior vertebral body tethering

## Abstract

VATS (video assisted thoracoscopic surgery) is routinely and successfully performed in minor and major complex thoracic procedures. This technique has been recently introduced for the treatment of severe forms of idiopathic scoliosis (IS) with the aim to repair the deformity, reduce morbidity and to prevent its progression in patients with skeletal immaturity. This study aims to present VATS in anterior vertebral body tethering (AVBT) approach to support the pediatric orthopedic surgeons during vertebral body fixation. Surgical and anesthesiologic tips and tricks are reported to assure a safe procedure. The study includes preadolescents with IS and a grade of scoliosis >40° that had a high probability of deterioration due to remaining growth (December 2018 to April 2021). Skeletal immaturity of enrolled patients was assessed by Sanders classification and Risser sign. Patients had a Risser score between 0 and 1 and a Sanders score >2 and <5. AVBT technique using VATS was performed by a senior pediatric surgeon assisting the pediatric orthopedic surgeon. Twenty-three patients have been submitted to VATS AVBT in the period of study (age range 9–14 years). The patients had a classified deformity Lenke 1A or B convex right and all types of curves were treated. In all patients, the vertebrae submitted to tethering surgery ranged from D5 to D12; mean curve correction was 43%. Three postoperative complications occurred: one late postoperative bleeding requiring a chest tube positioning on 12th postoperative day; one screw dislodged and needed to be removed; one child showed worsening of the scoliosis and needed a posterior arthrodesis. Initial results of VATS AVBT in growing patients with spinal deformities are encouraging. An appropriate selection of patients and a pediatric dedicated multidisciplinary surgical approach decrease intraoperative complications, time of operation and postoperative sequelae and guarantee an optimal outcome.

## 1. Introduction

Idiopathic scoliosis (IS) affects up to 5% of children younger than 18 years [1]. It can be classified according to the age of onset: infantile IS affects children aged 0–3 years, juvenile IS includes patients from 4 to 9 years of age while adolescent IS refers to patients aged 10–16 years [2,3]. External bracing is applied in less severe cases, while posterior spinal fusion is the gold standard for advanced curves, although it has associated morbidity and can lead to reduced mobility [4,5,6]. Degenerative disc disease can be documented at long term follow-up after spinal fusion, mainly at the lumbar level, although literature is not conclusive at this regard [7]. Other promising surgical treatments for IS have also been described in the literature, including traditional growing rods or magnetically controlled growing rods, which can be lengthened using external remote controller on an outpatient basis, developed in the last decade but still burdened by high complication and failure rates [8,9]. Anterior vertebral body tethering (AVBT) is a relatively recent technique, initially described by Crawford and Lenke [10], that allows gradual curve correction by changing the shape of the vertebra, while preserving spine mobility, with minimal associated morbidity [11,12,13]. It consists of tethering the spine using staple and screw instrumentation with a cable connecting the screws together; the cable modifies the local forces acting on the vertebrae involved and functions as an early and progressive mechanism of correction of the deformity [12]. Potential early or late complications include bleeding, nerve injury, under/overcorrection, progression of scoliosis, screw migration and/or cord rupture. Spinal fusion or redo tethering may be necessary in some cases [11,12].

The advent of video-assisted thoracic surgery (VATS) has brought a real revolution in pediatric surgery. VATS is routinely and successfully performed in minor or major thoracic procedures and in complex cases. VATS approach to perform AVBT has been recently introduced for the treatment of severe forms of IS [11,14] and initial results of this approach are encouraging.

The aim of our study is to present our preliminary experience with VATS AVBT, described together with surgical tips and tricks to decrease the risk of intraoperative complications, the operation time and postoperative sequelae.

## 2. Materials and Methods

Our retrospective study included preadolescent patients affected by idiopathic scoliosis with a Cobb angle higher than 40° and lower than 60°, that had a high probability of deterioration due to remaining growth. The patients had a classified deformity Lenke 1A or B, convex right. Skeletal immaturity was assessed by Sanders classification and Risser score. Patients enrolled had a Risser score between 0 and 1 and a Sanders score between 2 and 5. All patients were under conservative brace treatment and showed a worsening evolution. Exclusion criteria were: skeletal maturity, syndromic conditions, left-sided curves.

The study was performed according to the Declaration of Helsinki. Informed written consent was obtained from the parents and/or legal guardian after receiving information about the study.

### 2.1. Preoperative Assessment

All patients underwent routine preoperative blood test examination according to the Hospital protocol, cardiac evaluation together with electrocardiogram and cardiac ultrasound. Chest and spine X-ray were also performed. A preoperative multidisciplinary case discussion was performed for each patient, involving orthopedic surgeons, pediatric thoracic surgeon, anesthetists, neurologists, radiologists and physiotherapists.

### 2.2. Operative Technique

#### 2.2.1. Set-Up

**Figure 1:**
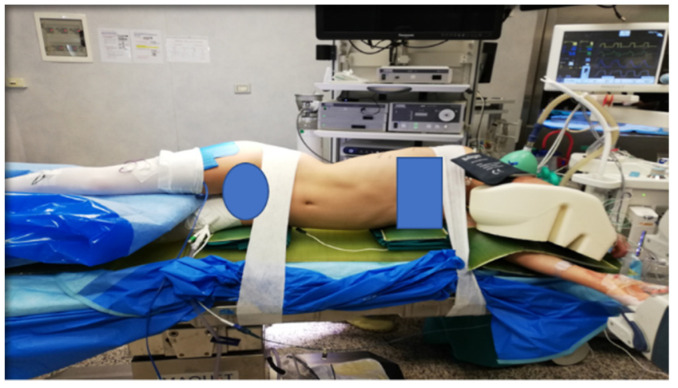
Position of the patient on the surgical table: the patient is positioned in left lateral 90° decubitus with the right side up. Appropriate soft gel pads are placed under the left shoulder and pelvis, in order to obtain a suspension of the spinal column with attenuation of the convexity. The patient is then secured with tape.

Standard VATS equipment is required. Under general anesthesia and intubation with double-lumen tubes for single lung ventilation, the patient is positioned in left lateral 90° decubitus with the right side up (all patients are affected by a convex right deformity). Appropriate soft gel pads are placed under the left shoulder and pelvis, in order to obtain a suspension of the spinal column with attenuation of the convexity. The patient is then secured with tape (Figure 1). 

**Figure 2:**
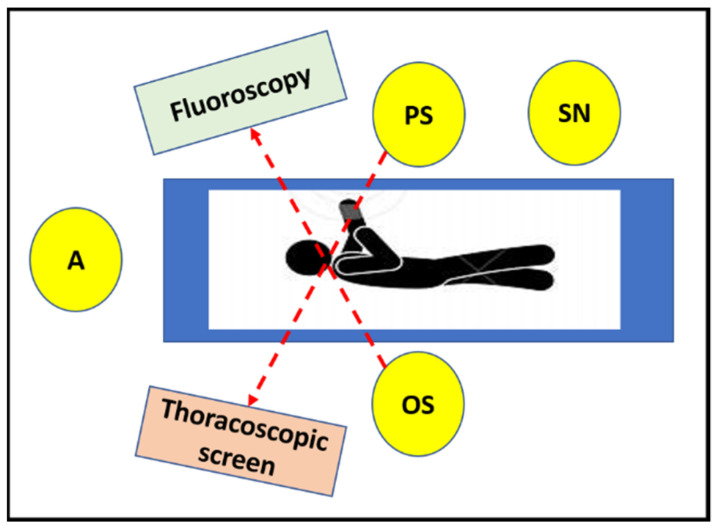
Position of the surgical team (scheme): the pediatric surgeon stands in front of the patient, with the thoracoscopic screen on the opposite side or at the bottom of the table, while the orthopedic surgeon stands on the back of the patient (in front of the spine) looking at the monitor of C-arm fluoroscopy. The scrub nurse stands on the left of the pediatric surgeon. (A = anesthetist, PS = pediatric surgeon, OS = orthopedic surgeon, SN = scrub nurse).

The pediatric surgeon stands in front of the patient, with the thoracoscopic screen in front of him on the opposite side of the table, while the orthopedic surgeon stands on the back of the patient (in front of the spine) looking at the monitor of C-arm fluoroscopy. The C-arm is kept under the operating table for the entire procedure. The scrub nurse stands on the left of the pediatric surgeon (Figure 2). The thoracoscopic column has a second articulated monitor which, positioned at the patient’s head, allows both the orthopedist and the scrub nurse to follow the thoracoscopic time.

Neuromonitoring is performed during the preoperative phase and throughout the tethering procedure with sensitive evoked potential (SEP) and motor evoked potential (MEP). To allow correct interpretation of evoked potentials, totally intravenous anesthesia is required. An erector spinae plane (ESP) block is performed under ultrasound guide before start of procedure.

Within 60 min before start of procedure, perioperative prophylaxis with Cefazolin 25 mg/kg is started.

#### 2.2.2. Surgical Technique

**Figure 3:**
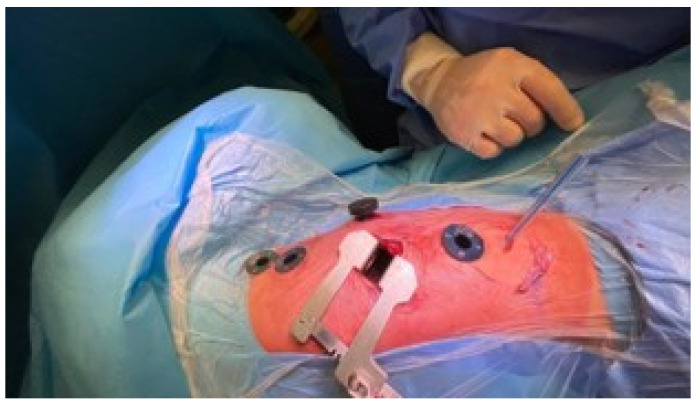
Incisions and position of the instruments: a small muscle-sparing thoracic incision is performed at the eighth intercostal space. Three 5-mm ports on the anterior axillary line between the fourth and the eighth intercostal spaces are inserted, at the same level as the vertebral defects as confirmed by fluoroscopy; one 10-mm port for a 30° camera is placed medially to the minithoracotomy.

A small muscle-sparing thoracic incision (5 cm in length) for instrument placement is performed at the 8th intercostal space. The trocar positioning includes: three 5-mm ports on the anterior axillary line between the fourth and the eighth intercostal spaces, at the same level as the vertebral defects as confirmed by fluoroscopy; one 10-mm port for a 30° camera is inserted medially to the minithoracotomy (Figure 3).

**Figure 4:**
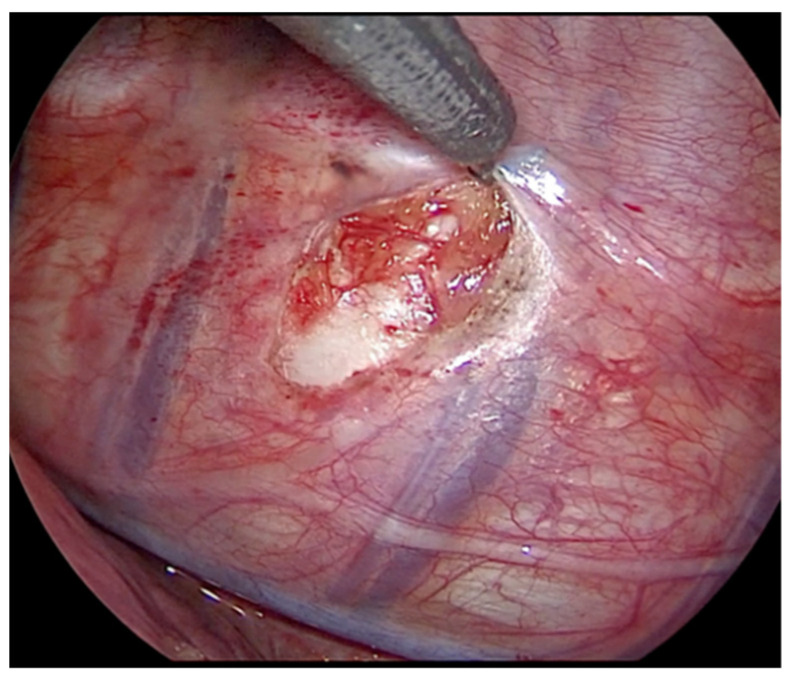
Thoracoscopic view: incision of the parietal pleura, lateral to the vertebral bodies and anterior to the rib heads.

**Figure 5:**
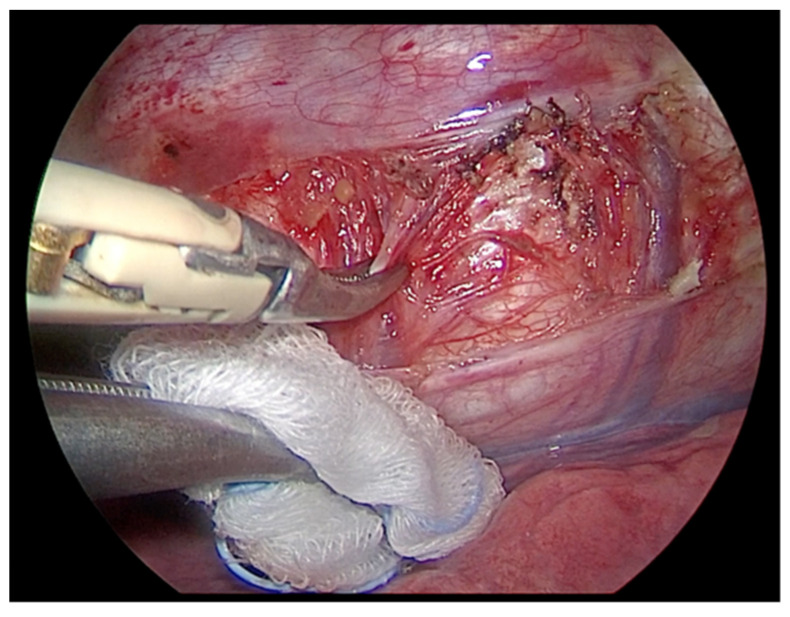
The intercostal vessels are identified and carefully dissected with bipolar forceps. Dissection and coagulation of the vessels below the pleura is helped through the mini-thoracotomy access with a pad mounted on Kelly instrument. This maneuver helps to keep the field clean and, in case of possible bleeding, to be faster in hemostasis.

The right lung is excluded, thus allowing a gasless procedure. The thoracic duct is visualized and preserved. The parietal pleura is incised lateral to the vertebral bodies, and anterior to the rib heads in a sequential fashion along the length of the curve (Figure 4). The intercostal vessels are identified and carefully dissected with bipolar forceps, or monopolar hook and harmonic scalpel. Dissection and coagulation of the vessels below the pleura is helped through the mini-thoracotomy access with a pad mounted on Kelly instrument. This maneuver helps to keep the field clean and, in case of possible bleeding, to be faster in hemostasis (Figure 5). Particular attention should be paid in the treatment of the segmental vessels near the azygos vein, due to the risk of severe bleedings.

**Figure 6:**
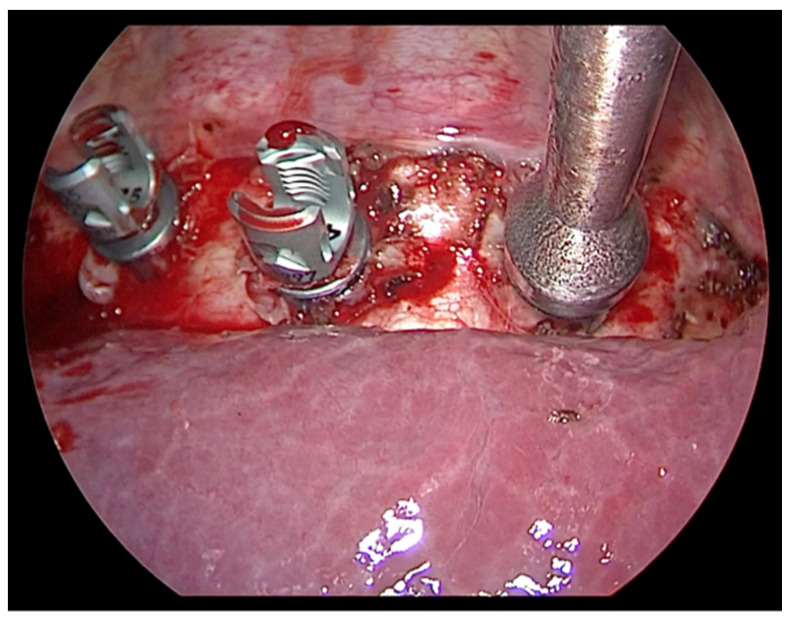
Vertebral bodies are instrumented with hydroxyapatite-coated screws and pronged staples.

**Figure 7:**
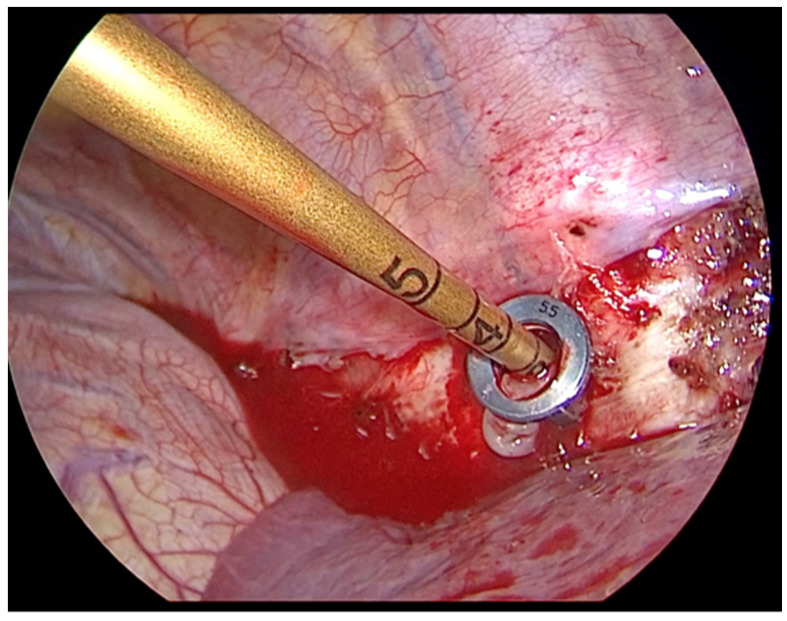
Insertion of the screws must take place perpendicularly to the vertebral body.

**Figure 8:**
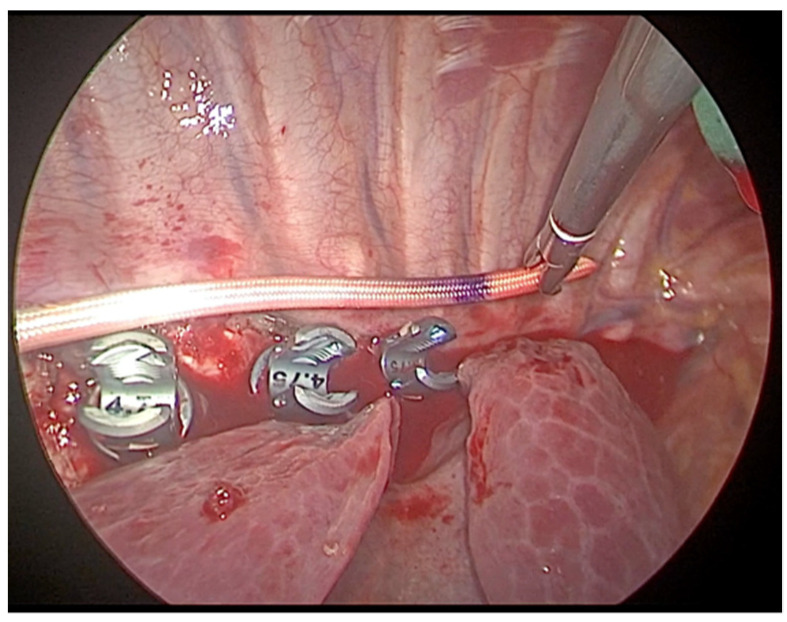
After all vertebral bodies are instrumented, the tether is inserted through the most caudal incision and placed in the tulips of the screws starting from the two most cephalic positions.

Vertebral bodies are instrumented with hydroxyapatite-coated screws and pronged staples (Figure 6). Insertion of the screws must take place perpendicularly to the vertebral body (Figure 7); the site of thoraco-port can be moved along in the intercostal spaces above and below to allow the orthopedic surgeon the adequate screw insertion. The screw positioning starts in the central part of the curve, then proceeds to the cranial vertebrae and subsequently to the caudal ones. To allow a good exposition of the spine, the diaphragm is pushed down with a swab through the mini-thoracotomy. The correct positioning of the screws is checked under fluoroscopy, both in the antero-posterior and lateral view. After all vertebral bodies are instrumented, the tether is inserted through the most caudal incision and placed in the tulips of the screws starting from the two most cephalic positions (Figure 8) and locked with screw set. The tether is put under traction with a dynamometric set until 300 Newton and progressively positioned through the more distal screws before completing the tethering of the whole spinal curve. The tether is cut proximally and distally, and a 12-French chest tube for drainage is inserted on the anterior axillary line into one of the trocar sites, then positioned along the costovertebral sinus, with its tip towards the apex to collect possible spillage of postoperative pleural effusion.

Lung recruitment is visualized by the cameraman to assist the anesthetist in achieving complete lung re-expansion.

Before trocar removal and suture of the surgical breaches, the intrapleural paravertebral intercostal nerve block is infiltrated with 10 mL of Mepivacain.

A chest X-ray is performed at the end of the procedure to document the position of the inserted prostheses and lung expansion.

### 2.3. Postoperative Management

During the first 24 postoperative hours, the patients are monitored in the intensive care unit, mainly for pain management. Mobilization is started from the first postoperative day, under the guidance of an expert physiotherapist. The thoracic drainage is kept in place until the daily output is less than 20 mL, then removed after performing a control chest X-ray.

## 3. Results

From December 2018 to April 2021, 23 patients with IS have been enrolled according to the diagnostic criteria and submitted to AVBT technique using VATS. The age range was between 9 and 14 years; data from nineteen females and four males were recorded.

All types of curves were treated and in all patients the vertebrae submitted to tethering surgery ranged from D5 to D12. Mean preoperative Cobb angle was 56.5° (range 33.0–79.0°). Mean duration of surgery was 250 min (range 220–440 min). Intraoperative blood loss was <150 cc (range 80–450). MEP and SEP monitoring have always been stable during surgery. No surgical nor anesthesiologic intraoperative complications were recorded. The thoracic drainage was kept for a median of 2 days (range 2 to 5 days). Patients were discharged on fifth to nineth post-operative day (median—6th day). Three postoperative complications occurred (3/23, 13%): one patient presented at the emergency department 6 days after discharge (12th postoperative day) with a pleural effusion and needed a chest tube positioning, with a subsequent uneventful hospital stay; in one patient a screw dislodged 5 weeks after the procedure and needed to be removed; one child showed a worsening scoliosis at long term follow-up and needed a posterior arthrodesis, performed 14 months after the initial procedure.

**Figure 9:**
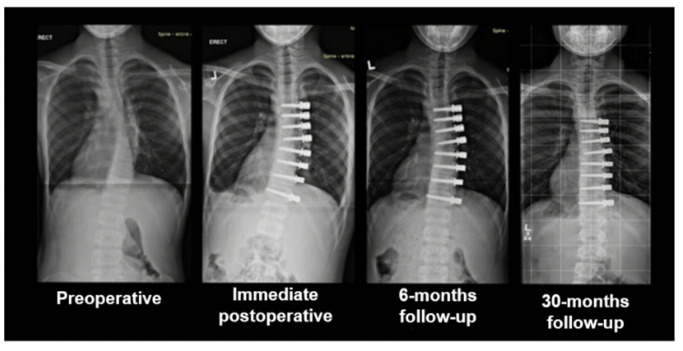
Evolution of the spine curve from the preoperative aspect to long-term follow-up.

Median postoperative follow-up ranged from 10 to 30 months. Mean immediate postoperative Cobb angle was 32° (range 20.0–56.0°), with a mean immediate curve correction of 43% (Figure 9). Mean follow-up Cobb angle was 37° (15.0–58.0°) at 6 months and 37° (12.0–45.0°) at 12 months, with a mean curve correction of 34.5% at both timepoints. All patients are well and free of sequelae at long-term follow-up.

## 4. Discussion

External braces for early disease or spinal fusion surgery are the conventionally proposed technique for the treatment of idiopathic scoliosis in childhood [11]. Good results of spinal fusion are reported in terms of curve correction, although the reduced motility of the spine leads to a limitation of the growth of the processed segments [4,5,6]. Possible degeneration of adjacent segments is reported as the most redoubtable risk of this technique. The technique of vertebral body tethering was designed to avoid these disadvantages. Although other recently described techniques seem promising in the management of IS, such as traditional growing rods or magnetically controlled growing rods, which are designed for the growing body and allow the correction to be modulated even in an outpatient setting, there are still many aspects that have to be adequately investigated and further studies are needed [8,9]. Minimally invasive surgery (MIS) continues to grow in popularity in all fields of pediatric surgery, including in the treatment of vertebral spine. VBT creates a relative correction of the curve giving a compression on the convexity of the column and a distraction on the concave side [15]. The curves gradually decrease, in accordance with the “Hueter–Volkmann law” [16]. In patients with residual skeletal immaturity, improvement of the defect derives from the possibility of developing the epiphyseal growth nucleus. This is why the ideal patient who can benefit from the fusionless technique as an alternative of spinal surgery is the pre-adolescent child. Thoracoscopic approach has demonstrated improved perioperative outcomes, decreased pain, and all benefits in term of morbidity, hospital stay and cosmetic results. This approach is becoming increasingly common, due to the well-known benefits of VATS, including a dramatic advance in visualization with magnified images. Although its critical weaknesses due to the two-dimensional visualization along with restricted maneuverability, VATS procedure for VBT allows a multidisciplinary access to the spine and the close collaboration between pediatric thoracic surgeon and orthopedic surgeon makes this procedure safe and feasible.

Our institution represents a national reference center for surgical treatment of the different forms of scoliosis. It is also a third level center for the application of the most modern minimally invasive surgical techniques. Over the past ten years, approximately 500 patients with scoliosis due to various conditions have been surgically treated. The use of thoracoscopy, however, imposed a different approach, with a technical innovation that required not only a multidisciplinary approach but also the establishment of a new learning curve by the entire team. Indeed, VATS procedure for VBT requires excellent teamwork and thoracoscopic expertise, and tip and tricks are required for a good result.

### Pitfalls, Tips and Tricks

- Adequate trocars positioning is essential in order to correctly place the screws in the vertebral bodies. Fluoroscopy use in anterior and lateral view guides the orthopedist to the correct insertion.
- The risk of bleeding is possible especially near the Azygos vein. The vascular dissection must be delicate and precise, using a bipolar forceps or a thin instrument combined with a bipolar coagulation device. The parietal pleura must be incised carefully along the marginal spine and a good exposure of the veins must be achieved. Small branches of the paravertebral veins need accurate hemostasis.
- Lesion of the phrenic nerve must be avoided, through a proper exposure and visualization of its course.
- The thoracic duct must be soon identified and avoided.
- Keep in mind any possible anatomic variations of the vascular, nerve and thoracic duct conformation and course.
- A correct interaction between pediatric and orthopedic surgeon, integrated by the combined use of thoracoscopic visualization and fluoroscopy, allow the orthopedist to correctly position the devices at the level of the vertebrae to be treated.
- The presence of a pediatric surgeon with advanced skills in thoracoscopic technique is required. This type of procedure should only be carried out in a third-level center where different professionals are present: expert radiology staff, pediatric orthopedist, pediatric thoracic surgeon, pediatric anesthetist, neurologist, expert physiotherapist.

Our results seem consistent with the previous experiences of AVBT reported in the literature so far, with a 13% of complication/reoperation rate and a mean curve correction of 43%, that are similar to other series [4,17,18]. The thoracoscopic set-up and time did not affect the duration of surgery, with a mean duration of 250 min that results comparable to other published experiences [18].

Limitations of our experience are the small number of cases treated so far, the lack of a prospective control group and a relatively short follow-up time.

## 5. Conclusions

AVBT is an innovative technique in pediatric orthopedic surgery, which allows to accompany the growth of the spinal column in the preadolescent patient. Application of VATS for the treatment of this kind of deformity is a new era for further development. The thoracoscopic visualization allows to facilitate the work of the orthopedist and the minimally invasive approach accelerates the mobilization and the postoperative course, with advantages in terms of postoperative pain and cosmesis. Surgical tips and tricks are mandatory to decrease the risk of intraoperative complications and postoperative sequelae. The multidisciplinary approach guarantees a safe and complete management of these patient.

## Data Availability

Data reported in this study are available upon request from the corresponding author.
